# Ibuprofen-Induced Toxic Epidermal Necrolysis: Report of a Fatal Case

**DOI:** 10.7759/cureus.91001

**Published:** 2025-08-25

**Authors:** Esperanza Vanessa Matadamas Carmona, Marco Antonio Jimenez Ferreira

**Affiliations:** 1 Internal Medicine, Hospital General de Acapulco, Acapulco, MEX; 2 Pathology, Hospital General de Acapulco, Acapulco, MEX

**Keywords:** cutaneous adverse drug reaction, ibuprofen, intravenous immunoglobulin, sjs, ten, toxic epidermal necrolysis

## Abstract

Ibuprofen is a widely used and easily accessible medication that belongs to the nonsteroidal anti-inflammatory drug class. Its most common adverse effects are gastrointestinal. However, there are a few reported cases of fatal side effects.

We report a case of toxic epidermal necrolysis (TEN) potentially affecting more than 80% of the total body surface area, triggered by ibuprofen in a 77-year-old female patient after taking it for the first time. The diagnosis of TEN was confirmed through skin biopsy.

The severe mucocutaneous reactions, Stevens-Johnson syndrome and toxic epidermal necrolysis, are characterized by necrosis and skin sloughing. Both share the same pathophysiology; however, they differ based on the total body surface area affected. Toxic epidermal necrolysis is defined as an affected body surface area greater than 30%, so early diagnosis and identification of the cause are crucial to determine its course and prognosis.

## Introduction

Stevens-Johnson syndrome (SJS) and toxic epidermal necrolysis (TEN) are dermatologic emergencies characterized by generalized epidermal necrolysis with sloughing. They are considered to have the same pathophysiology and are classified based on total body surface area (TBSA) [1]. A TBSA >30% defines TEN. TBSA between 10% and 30% is defined as TEN/SJS overlap [2].

Stevens-Johnson syndrome/toxic epidermal necrolysis (SJS/TEN) typically begins with a prodrome of fever and flulike clinical manifestations lasting 1 to 3 days, followed by photophobia, conjunctival itching, dysphagia, and skin tenderness. Typical lesions are so-called dusky atypical targets, in which the central component of an erythematous macule or papule is dull or dusky, signifying epidermal necrosis and death. Individual targetoid papules and larger coalescing areas of dusky erythema evolve over 1 to 3 days into vesicles, bullae, or widespread denuded skin [3].

In adults, the disease typically occurs more in women between 40 and 50 years of age, and its incidence increases with age. In 85-90% of cases, epidermal necrolysis is drug-induced and usually occurs within four to 28 days (with a maximum of 56 days) of drug ingestion (for the first time in life) [4].

The disease evolves in two phases: the acute phase is sudden and can be life-threatening depending on the extent of cutaneous and mucous membrane lesions. Respiratory and septic complications determine the prognosis. In the chronic phase, it is accompanied by multiple sequelae in almost 90% of patients, impacting quality of life. Physical sequelae mainly involve the skin (dry skin, abnormal pigmentation, extensive scarring, nail abnormalities, skin pain), eyes (dry eye, chronic conjunctivitis, trichiasis, symblepharon, corneal abnormalities with vision disturbances leading to blindness), mouth (dry mouth, dental abnormalities), genitals (preputial and vaginal synechiae), digestive tract (esophageal stricture), and bronchi (bronchiolitis obliterans) [4]. Mortality from TEN is high, and the main cause is sepsis [5].

## Case presentation

A 77-year-old female patient with a history of controlled hypertension presented with pain in her feet. She attended a private clinic where she was prescribed oral ibuprofen (800 mg tablet, single dose, first time taken). Six hours later, she presented with generalized erythema, intense burning sensation, pruritus, and multiple maculopapular rash beginning on the back and gluteal region, with fever up to 42°C. Over the following days, the symptoms progressed to generalized blister formation and sloughing of the skin. Blisters were flaccid, with clear fluid, located on the back, posterior legs, and buttocks, with erythema. On the oral and nasal mucosa erosions there were deep fisures, some of them covered with hematic crusts. She presented to the emergency department 10 days after the onset of symptoms. On admission, the patient presented with clinical signs of respiratory distress and was initiated on deep sedation, analgesia, and invasive mechanical ventilation.

**Figure 1:**
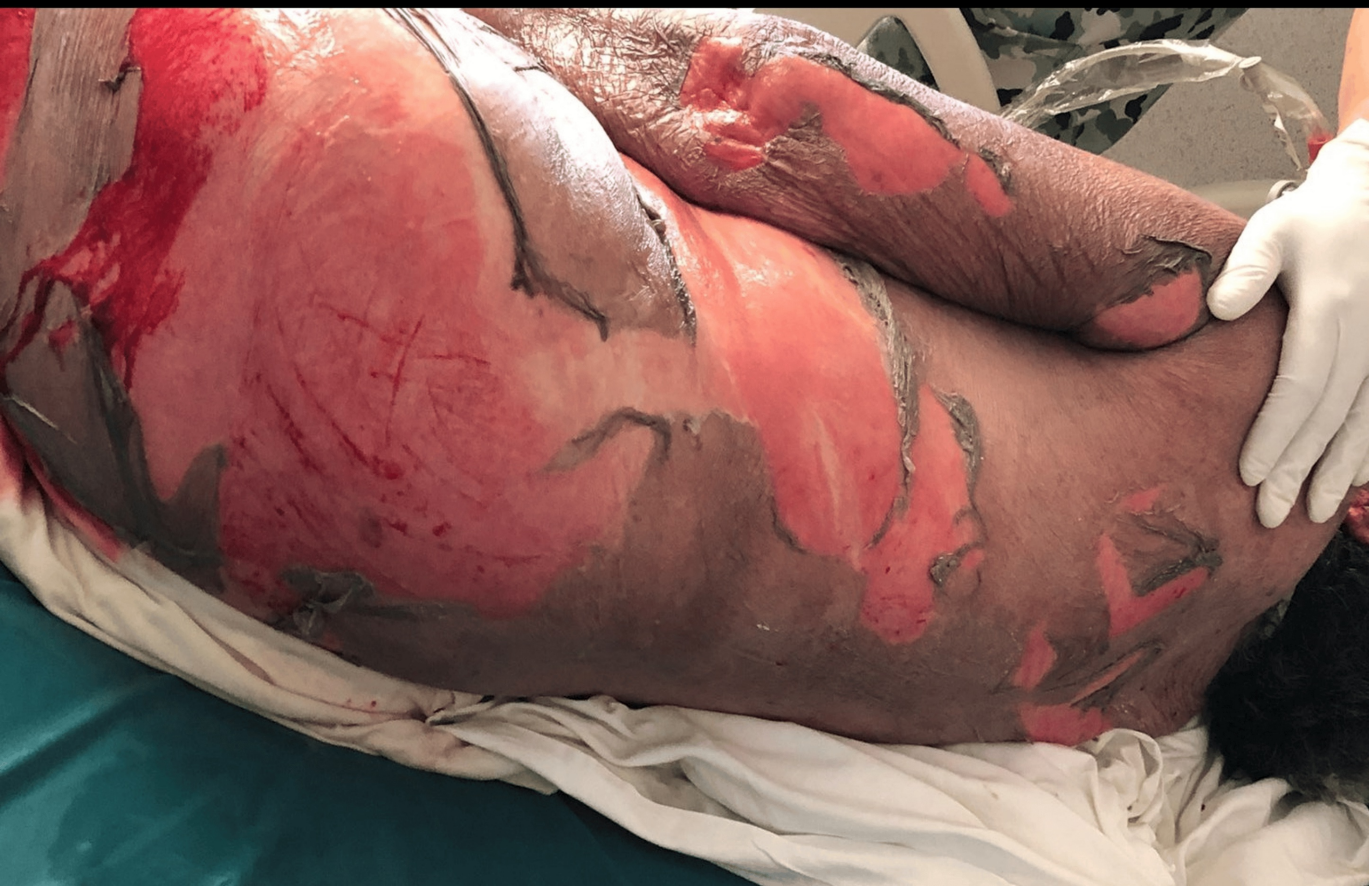
Epithelial detachment in the trunk and back area.

**Figure 2:**
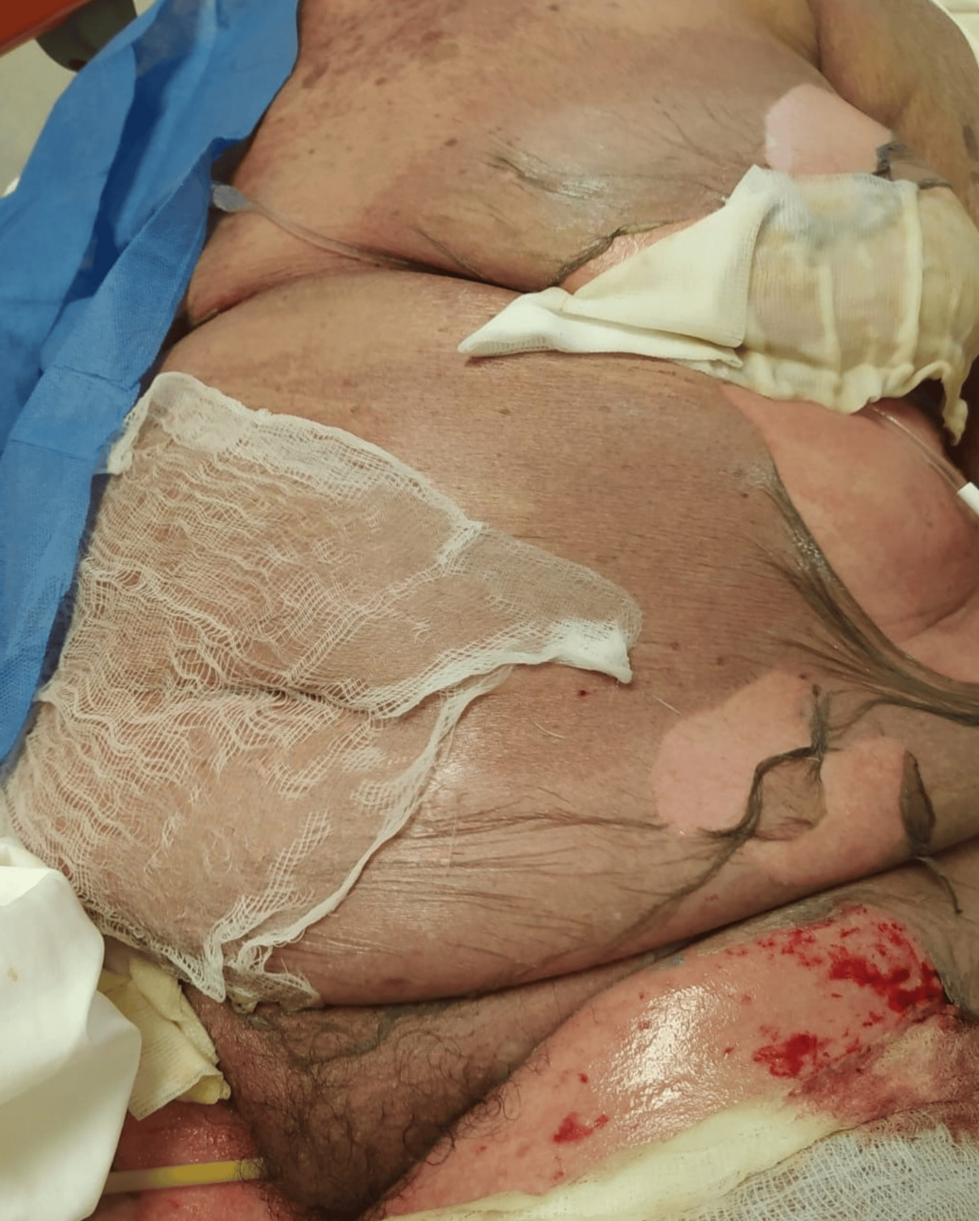
Denuded areas predominantly on the breast and back.

**Figure 3:**
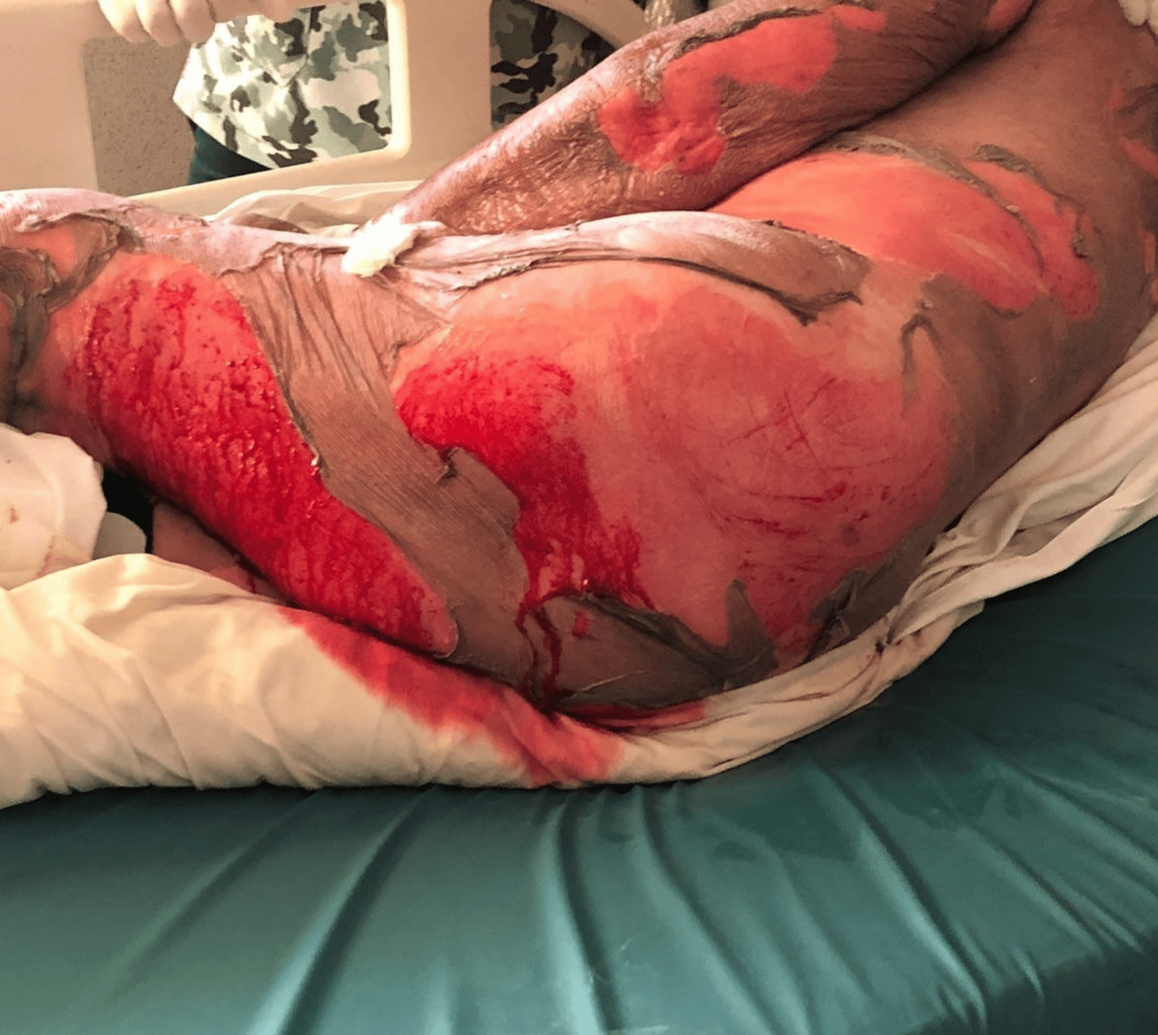
Denuded areas and bleeding on the posterior region of the thigh.

**Figure 4:**
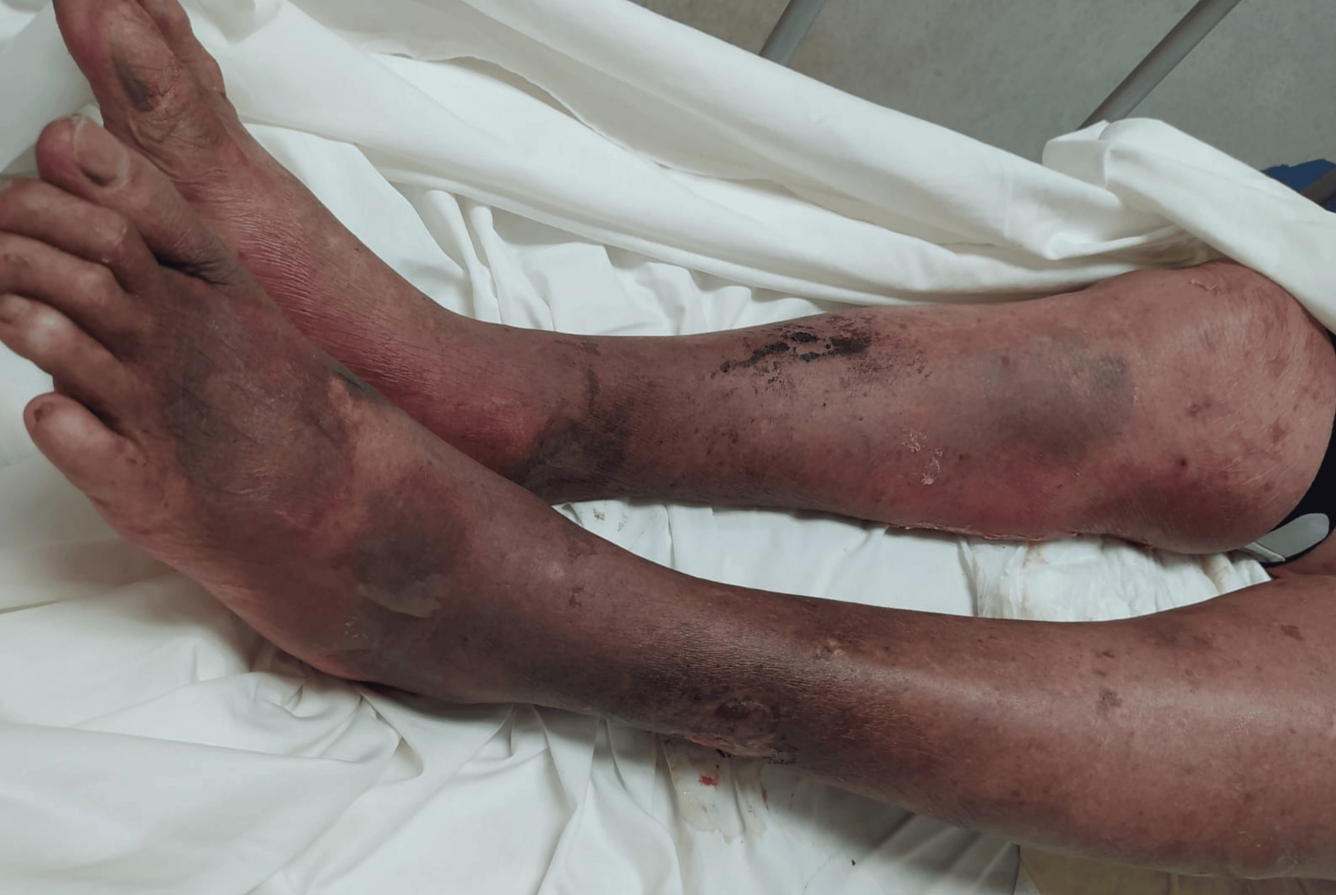
Diffuse erythema with hyperpigmented spots on distal extremities.

Physical examination revealed a generalized dermatosis with a disseminated mucocutaneous reaction covering more than 80% of the patient's total body surface area (Figure 1). On the face, diffuse erythema was observed with a periorificial tendency, predominantly periorbital and perioral, consisting of hematic and meliceric crusts. On the trunk, the area was affected with denuded areas with hyaline exudate, predominantly in the breasts and back (Figure 2), and bleeding in some areas (Figure 3). On the distal extremities, there was diffuse erythema with poorly defined dark brown hyperchromic spots without the presence of denuded areas (Figure 4).

Laboratory exams were performed, including complete blood count (CBC), liver function tests (LFTs), renal function tests (RFTs), and serum electrolytes. The results of these exams are presented in Table 1.

**Table 1:** Blood workup of the patient. HCT: Hematocrit; AST: Aspartate aminotransferase; ALT: Alanine transaminase; LDH: Lactate dehydrogenase; BUN: Blood urea nitrogen; CK MB: Creatine kinase MB; Na⁺: Sodium; K⁺: Potassium; Cl⁻: Chloride.

	Patient value	Reference range
Hemogram		
Hemoglobin	9.2	12-16 g/dL
HCT	27.5	37-47%
WBC count	6.3	3.4 - 9.7x10^9^/L
Platelets	329	158-424x10^9^/L
Liver Function Tests		
Total bilirubin	0.8	0.40-1.30 mg/dL
Direct bilirubin	0.3	0.0-0.35 mg/dL
Indirect bilirubin	0.5	0.20-0.80 mg/dL
Total protein	5.2	6.4-8.3 g/dL
Albumin	2.2	3.5-5.2 g/dL
AST	20	8-40 U/L
ALT	18	5-35 U/L
LDH	290.5	200-400 U/L
Glucose	103	72-116 mg/dL
Renal Function Tests		
BUN	91	9.3-18.6 mg/dL
Creatinine	6.4	0.7-1.2 mg/dL
Creatine Kinase	551	26-140 U/L
CK MB	42.7	<25 U/L
Serum Electrolytes		
Na⁺	136	135-152 mEq/L
K⁺	6.4	4.10-4.50 mEq/L
Cl⁻	106	95-109 mEq/L

A biopsy was taken from a lesion located on the anterior aspect of the right thigh and sent to the Pathology Department.

**Figure 5:**
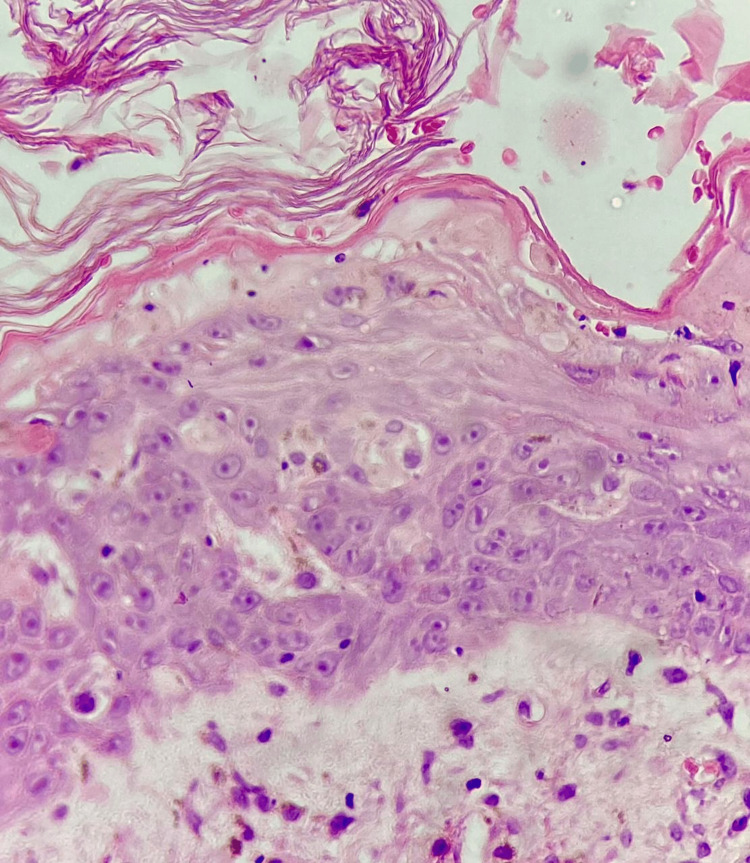
A histological section with hematoxylin-eosin in which the stratum corneum is observed as a basket weave-like, epidermic keratinocytes with spongiosis and acantholysis.

**Figure 6:**
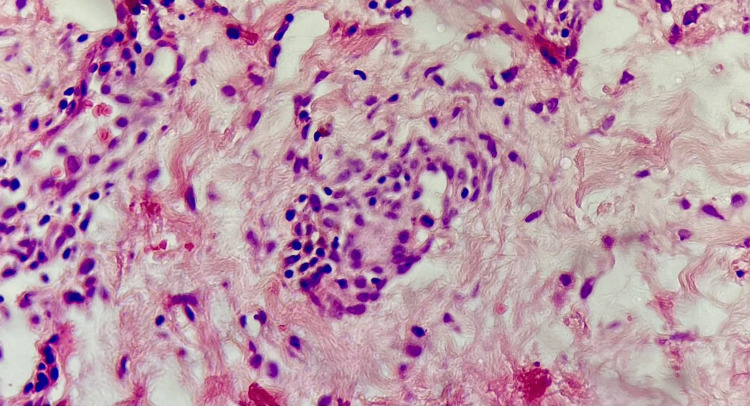
Vascular damage is observed with little inflammatory infiltrate, predominantly lymphocytic.

The histopathological study was collected (Figure 5 and Figure 6), and the diagnosis of toxic epidermal necrolysis was established.

Treatment was initiated with immunoglobulin at a dose of 400 mg/kg/day (85 kg x 400 mg = 34,000 mg per day) in vials of 6 g/120 ml immunoglobulin (34,000 mg = 34 g/6 g/120 ml = 6 vials per day for five days), requiring a total of 30 vials to cover the 5-day treatment. Vaseline-coated compresses were applied to all denuded areas. Unfortunately, despite the treatment, the patient's condition worsened, leading to multiple organ failure and death.

## Discussion

Among the serious adverse drug reactions are Stevens-Johnson disease and toxic epidermal necrolysis, both characterized by significant necrosis and skin sloughing. Their difference lies in the percentage of body surface area affected.

It should be noted that the use of certain commonly used nonsteroidal anti-inflammatory drugs, as seen in this case, may carry a risk of severe complications that can lead to multiple organ failure and subsequent death. Several risk factors associated with increased mortality have been identified, including increasing age, racial or ethnic identity, chronic kidney disease, pneumonia, sepsis, and malignancy [5].

SJS/TEN is a disease spectrum with high morbidity and mortality; previous studies have reported mortality rates for SJS to be around 19.4% to 29% and for TEN to be around 14.8% to 48% [6].

Stevens-Johnson syndrome and toxic epidermal necrolysis have high mortality rates, with risk factors for mortality identified in the severity-of-illness SCORe of Toxic Epidermal Necrolysis (SCORTEN) [6].

Known risk factors associated with mortality include age older than 40 years, the presence of associated malignancy, TBSA involvement >10%, serum bicarbonate <20 mmol/L, serum urea nitrogen >10 mmol/L, serum glucose >14 mmol/L, heart rate >120 beats per minute, and chronic kidney disease [6].

While SJS/TEN is relatively rare, it is associated with high mortality and directly correlates with the BSA involved, increasing from SJS to SJS-TEN overlap syndrome to TEN [6].

The pathogenesis of SJS/TEN is not yet fully understood, but clinical and histopathological studies indicate that they are types of delayed hypersensitivity reactions primarily triggered by drugs (95%). Infections represent the second major cause. Common clinical triggers include antibacterial, antiepileptic, antipyretic analgesics, antigout medications, biologics, and traditional Chinese medicines, with pathogens like Mycoplasma and herpes simplex virus also being implicated [7].

In conclusion, IVIG combined with corticosteroid treatment shortens the recovery time of SJS/TEN patients. Furthermore, IVIG therapy is likely to reduce the mortality of this disease. However, this outcome needs more high-quality studies to be confirmed [8]. 

## Conclusions

Ibuprofen is a very commonly used medication due to its anti-inflammatory, antipyretic, and analgesic effects. With the increase in patient longevity, there has been an increase in the use of these medications to control pain and inflammation in chronic rheumatic or degenerative diseases. The main mechanism of this medication is the nonselective inhibition of the cyclooxygenase enzyme, thereby altering the synthesis of arachidonic acid into prostaglandins, prostacyclin, and thromboxanes. The use of ibuprofen is very safe with few side effects, mainly gastrointestinal. However, in rare cases, fatal adverse effects have been reported.

It is important to make an early diagnosis of the disease and identify the causative agent. In the case of drug-related causes, it is important to identify and discontinue the drug immediately, preventing the condition from worsening. Toxic epidermal necrolysis is a very serious disease that requires comprehensive management involving several services in an intensive care unit or burn unit. Therefore, early diagnosis with identification of the causative agent, staging of disease severity from the moment the patient is admitted, monitoring of progression, and optimal and effective treatment will improve the prognosis and prevent sequelae.
